# The Role of the Private Sector in Supporting Malaria Control in Resource Development Settings

**DOI:** 10.1093/infdis/jiaa488

**Published:** 2020-10-29

**Authors:** Robert T Jones, Lucy S Tusting, Hugh M P Smith, Sylvester Segbaya, Michael B Macdonald, Michael J Bangs, James G Logan

**Affiliations:** 1 Arthropod Control Product Test Centre, London School of Hygiene & Tropical Medicine, London, United Kingdom; 2 Department of Disease Control, London School of Hygiene and Tropical Medicine, London, United Kingdom; 3 Johns Hopkins Center for Communication Programs, Accra, Ghana; 5 lnternational SOS, Ltd., Timika, Papua Province, Indonesia; 6 International SOS, Ltd., Kolwesi, Lualaba Province, Democratic Republic of Congo

**Keywords:** private sector, malaria, vector-borne disease, resource development setting

## Abstract

Industrial operations of the private sector, such as extraction, agriculture, and construction, can bring large numbers of people into new settlement areas and cause environmental change that promotes the transmission of vector-borne diseases. Industry-related workers and communities unduly exposed to infection risk typically lack the knowledge and means to protect themselves. However, there is a strong business rationale for protecting local resident employees through integrated vector control programs, as well as an ethical responsibility to care for these individuals and the affected communities. We discuss the role and challenges of the private sector in developing malaria control programs, which can include extensive collaborations with the public sector that go on to form the basis of national vector control programs or more broadly support local healthcare systems.

Large-scale natural resource extraction, extensive agriculture, and other industrial projects can generate wide-ranging social impacts, and have historically been major drivers of disease epidemics, including those of sexually transmitted diseases, human immunodeficiency virus (HIV), and tuberculosis [1, 2]. However, industrial activities also have significant environmental impacts, which, when coupled with changes in demography, allow for increased transmission of vector-borne diseases (VBDs) [3, 4].

In Brazil, for example, deforestation and the inadvertent creation of pools of standing water in road ditches, dams, mining pits, and vehicle ruts, provide suitable breeding habitats for the main vectors of malaria in the Amazon, *Anopheles darlingi* [5]. These mosquitoes are notably more abundant in altered landscapes, and the provision of habitats beneficial to their breeding, combined with an influx of immunologically naive migrant populations, has allowed for spikes in malaria cases at development sites [5, 6]. In Thailand, the establishment of rubber plantations has influenced local malaria transmission dynamics through changes and variations of vector species composition, abundance, and blood-feeding behaviors [7]. Activities related to agriculture, forestry, mining, and highway and hydroelectric dam construction also bring people into contact with the sand fly vectors of leishmaniasis [8], and in parts of Australia, flooded mine shafts have created breeding sites for the mosquito vectors of dengue viruses [9].

It is clear, therefore, that private sector activities can be associated with increases in vector habitats and increased exposure to VBDs, and it is reasonable to expect that this will become more of an issue in the future as demands for agricultural, energy, and mineral commodities rise. In some cases, private organizations involved in these activities have, in the past, made contributions to the control of VBDs. In the 1920s, employers in Bolivia were required to distribute quinine to their employees free of charge in areas where malaria was prevalent, and if involved in agricultural, industrial, and commercial pursuits, take measures to protect dwellings from mosquitoes [10]. At the same time in Zambia, copper mines invested in environmental management interventions to sustain healthy labor forces [11].

Today, significant investments in environmental and health impact assessments that assist in devising site-specific risk management plans imbue a sense of corporate responsibility in industrial sectors to support environmental, health, education, and socioeconomic programs in the communities that they directly and indirectly impact [12, 13]. Depending on location and circumstances, coverage should extend to protection from VBDs, and include community engagement and social outreach, greater health awareness, and expanded vector control activities in order to mitigate the potential negative aspects of development in the area [14, 15]. The private sector, therefore, can have an important role in both creating and mitigating problems associated with VBD risk. Whilst national and local governments often form the health policy umbrella under which other partners must operate, there is growing involvement of the private sector in healthcare and preventive medicine endeavors within the framework of a multisectoral approach [16]. These approaches also expand to stakeholder engagement on access to education, improved infrastructure, small business creation, and other projects. Here, we review the powerful and efficient role that the private sector can play in supporting malaria control in resource development settings.

## METHODS

A literature search was performed using archives of published biomedical and life sciences journal literature available through PubMed (MEDLINE) and Web of Science. These searches were made without restrictions on languages or publication dates. Active searches were made in June–July 2017. Additional resources, such as reports from extraction companies and funding bodies, were subsequently accessed to find case studies and provide contextual information to the findings of the literature search. We focused on national and local partnerships between disease control programs and commercial organizations whose primary business is not related to vectors but whose operations ultimately expose their employees and impacted communities to VBDs.

## INVOLVEMENT OF THE PRIVATE SECTOR IN SUPPORTING VECTOR-BORNE DISEASE CONTROL

There are different approaches that private sector collaborative efforts have taken to meet the challenges of VBDs, including efforts that have resulted in the introduction of a new long-lasting mosquito net [17], and drug donation and distribution partnerships [18].

VBD control programs established by mining and other natural resource extraction enterprises are motivated to maximize productivity and contribute to a boarder corporate social responsibility strategy that creates a positive “social license” to operate in an area. At some point in the project development, control is redirected into integrated programs to extend its reach to the local community, which is affected by the project’s presence and is typically the primary source of project labor [15]. Disease control programs offered by the private sector can further serve as foundations for other health initiatives and can act as centers of excellence that provide platforms for capacity building.

The Philippines provides an example of a successful partnership between a multinational oil and gas company and the national government of the country in which it operates. Pilipinas Shell Foundation, Inc. (PSFI) launched a social investment program in 1999 that worked with the provincial government and the department of health to set up village laboratories for malaria testing in Palawan province, and to raise awareness of malaria prevention [19]. The success of the program led to PSFI receiving a grant from the Global Fund to Fight AIDS, Tuberculosis, and Malaria (GFATM) to expand its activities [20, 21]. Four years later, PSFI received additional funding to increase its coverage to 40 malaria-endemic provinces, followed by more funds in 2012.

The community health workers in Palawan who trained under the program provided early diagnosis and treatment, and their community awareness-raising activities strengthened the prevention practices of residents [22]. Although cases in Palawan have remained much higher than in other provinces [23], and it is difficult to disentangle the specific impact of the private sector, together the program’s activities reduced the malaria cases by 92% between 2005 and 2018 [24].

Pivotal to the accomplishments of the program was a wide range of approaches that incorporated social and economic factors, with an emphasis on capability building and strong stakeholder engagement. Local government units were engaged in the early stages of the program and provided transport for bed net distribution, incentives for community volunteers, travel of health personnel, office spaces, and venues for training. For their part, the municipal and provincial health offices provided technical assistance, monitoring, and other support. Overseeing the program and providing policy direction to all local government units, was the department of health [19].

Other examples of governments working closely with the private sector come from Brazil, where participation by a mining company operating in northern Amapá State in a public malaria control program (MCP) led to a significant reduction in malaria incidence and malaria-related morbidity and mortality [25], and Chad, where public-private partnerships form an important component of community health outreach programs [16]. In Equatorial Guinea, the Bioko Island Malaria Control Project was launched with funding from a consortium led by Marathon Oil Corporation and from the national government, and resulted in a major reduction in malaria transmission [26] (Table 1).

**Table 1.** Summary of Examples of Malaria Control Programs With Private-Sector Involvement

Sector	Company	Country	Program Summary	Reference
Agriculture	Illovo Sugar	Malawi	IRS	[30]
	Zambia Sugar	Zambia	IRS, malaria case management, IPTP, education and behaviour change communication	[27, 28]
Mining	Mineração Novo Astro S/A	Brazil	Vector control and surveillance services, investments in staff, provision of equipment	[25]
	AngloGold Ashanti	Ghana	IRS, bed net distribution, environmental management, insecticide resistance management, education, surveillance	[29]
	Société d’Exploitation des Mines d’Or de Sadiola	Mali	IRS, larviciding, breeding site removal, household malaria education	[31]
	Konkola Copper Mines, Mopani Copper Mines	Zambia	IRS, malaria case management, IPTP, education and behaviour change communication	[27, 28]
	Roan Antelope, Mufulira, Nkana‐Kitwe, and Nchanga mines	Zambia	IRS, malaria case management, IPTP, education and behaviour change communication	[11]
Oil and gas	ExxonMobil, Petronas, Chevron	Chad, Cameroon	Insecticide-treated bed nets, chemoprophylaxis among nonimmune workers	[16]
	Marathon Oil	Equatorial Guinea	IRS, bed net distribution, ACT introduced free of charge to children and pregnant women, IPTP, training of medical staff, communication campaign	[26]
	Shell	Philippines	IRS, insecticide-treated bed net distribution, diagnostic and treatment provision, capacity building	[19, 20]

Abbreviations: ACT, artemisinin-based combination therapy; IPTP, intermittent preventive treatment in pregnancy; IRS, indoor residual spraying.

Large extraction and other industrial companies have excellent logistics and networking capabilities, sufficient monetary resources, advanced systems in healthcare and public health, and the ability to develop close, long-term national and international relationships [15, 32]. Further, their often-remote locations, the immediate resources available to them, and their relationships with surrounding communities allow companies to respond quickly and appropriately to malaria and other disease outbreaks and install preventative measures in a timely and effective manner.

## BENEFITS TO NATIONAL AND STATE DISEASE CONTROL PROGRAMS

Public-private partnerships have helped the Sabah State MCP to overcome the barriers associated with protecting vulnerable populations working on plantations in remote geographic locations [33]. Such sites can attract workers from neighboring endemic countries, who either may import malaria or be at higher risk of infection; therefore, working directly with plantations, mining, and other industrial projects can ensure higher rates of coverage with indoor residual spray (IRS) and insecticide-treated nets.

The establishment of health services can also be an excellent way to reach mobile populations, and offering access to facilities at their place of work helps to build trust with the MCP. These static services can provide education related to disease awareness and prevention, and encourage workers to alert either the program or their employers when they are unwell. A further benefit for the MCP is that the plantations have financial and human resources that they can commit to malaria control, thus liberating state program resources to concentrate on areas where there is ongoing transmission outside of private sector coverage [33]. Some companies also provide land, buildings, and equipment, while others maintain their own clinics that are capable of diagnosing and treating malaria [34].

In addition, the private sector has more intangible assets to offer to vector control programs, such as project management skills, fiscal discipline and transparency, leadership skills and governance expertise, distribution capacity, and strategic and long-term planning capabilities [17, 35, 36]. Finally, private sector malaria control operations may provide valuable experience and best practices, and be a foundation for national program activities. For example, after a hiatus of 30 years, the Zambia national IRS program was restarted in 2003 with the technical support of Konkola and Mopani Copper Mines and Zambia Sugar, who had already been running successful IRS operations for many years [27, 28]. Likewise, the Ghana national IRS program grew from the foundation of the AngloGold Ashanti mining operations; while in Malawi, programs followed from the Illovo Sugar plantation operations in Nkotakota District [29, 30].

While engaging in control operations, private sector stakeholders can also provide a valuable role in overall vector-borne and other infectious disease surveillance by gathering and transmitting information into a centralized network to alert regional and international authorities of disease trends and outbreaks [37]. In more remote localities, disease-related data collected by such networks may represent the only reliable and up-to-date information from that area.

## BENEFITS TO THE COMMERCIAL SECTOR

There is a strong business case for investing in VBD programs, which ultimately have impacts on project profitability through reduced employee absenteeism and turnover, decreased health costs, and improved employee morale [35, 38, 39]. Disease control is a sound investment and insignificant cost compared to loss of productivity over time or outright failure of a commercial venture.

The Société d’Exploitation des Mines d’Or de Sadiola gold mine in the Kayes region of Mali benefitted tremendously from a 70% reduction in malaria cases within 2 years of reinstating IRS in their malaria vector control program. Spraying was part of a community-based drive, which also included a partnership for the distribution of bed nets, sponsored by the President’s Malaria Initiative, to reduce mortality and morbidity amongst mine employees and their dependents, and to improve health in the surrounding villages [31].

Another example of a successful private-private partnership occurred many decades ago in northern Zambia whereby 4 large copper mines implemented integrated malaria control in mining communities with a strong emphasis on environmental management of malaria vector mosquito habitats [11]. Together with access to rapid diagnosis and treatment, and the use of bed nets, these programs witnessed dramatic reductions in malaria incidence (baseline malaria incidence rate was reduced by 50%–75% in the first 3 years), resulting in a large number of work shift losses and absenteeism being averted.

Efforts to control malaria have now become part of the corporate social responsibility of many large industrial companies operating in malaria-endemic areas [40]. Corporate social responsibility is a management concept whereby companies integrate social and environmental concerns in their business operations and interactions with their stakeholders. In addition to helping private sector organizations to achieve economic targets, reducing malaria can help meet the expectations of shareholders and stakeholders by generating goodwill in the community.

Engagement with communities and partnerships with other organizations with similar objectives will increase the scale and impact of a disease management program [41–43]. Without active participation in the broader community and sector-wide activities, efforts led by companies will be limited in coverage. In most instances, the benefits of addressing diseases in the community exceed the small additional costs to a company of extending its health services into the surrounding populations [35].

## MUTUAL BENEFITS OF PARTNERSHIPS

Corporate investment in VBD control programs and the availability of their resources and infrastructure has allowed some companies to secure funding from external donors as not-for-profit legal subsidiaries, thereby allowing the scale-up of interventions that would not otherwise have been possible [44]. AngloGold Ashanti became the principal recipient of a GFATM grant to expand IRS to 40 districts in northern Ghana, and in doing so demonstrated that a successful public-private partnership project can have a great impact in the fight against malaria. Malaria cases in the Obuasi mine area were reduced by approximately 75% [29, 40].

Forming partnerships between private and public sectors allows for the pooling of limited resources and expertise, including funds, staff time, and local knowledge essential for success and sustainability. These collaborations present opportunities for leveraging such resources to maximize different organizational capabilities and expertise. Further, multilateral partnerships allow for a wider response coverage and perspectives when involving different types of organizations and sectors, improved understanding of community needs, and better access to those communities [35]. A crucial feature of partnerships in resource-limited settings is ensuring that there is little or no duplication of effort. Importantly, sharing lessons learned, and ultimately forming partnerships, can lead to more innovative and effective programs that improve support and services for those populations affected by disease. Moreover, there are opportunities to address diseases beyond those that are just transmitted by vectors. Addressing water and sanitation limitations within larger projects, for instance, can help to reduce diarrheal disease and outbreaks (eg, cholera), so the platform can be extended to encompass a wider range of public health issues [13, 14].

## BEST PRACTICES

The private sector remains a poorly understood and under-utilized resource for initial planning and implementation of VBD control programs or augmentation of existing public health services to improve efficiency. Experiences from collaborations between the Malaysian MCP and plantation companies in Sabah State to reduce the burden of malaria [33], in addition to those from the mining industry, suggest that future partnerships should be expanded and promoted. These partnerships must take place in a broader multisectoral approach led by the health sector. The World Health Organization’s Global Vector Control Response (GVCR) advocates for the establishment of national interministerial task forces for multisectoral engagement in vector control, and whilst the core decision-making function within these task forces resides with ministerial representatives, they should have representation from the private sector and other stakeholders [45]. Other best practices include: written agreement on specific, commonly defined goals for the partnership, negotiation of the division of responsibilities and resources to avoid redundant services, and the establishment of clear objectives and expectations between partners. The identification of appropriate people, expertise, and organizations to work with, agreement on shared activities for the program, and engagement of staff at all levels for supporting the local and regional/national program, are important [46].

The foundations of good programs should be evidence based; therefore, the directed use of surveillance, monitoring, and evaluation data to identify sites with local or imported cases, or which are at higher risk of outbreaks, is crucial, and there must also be sharing of morbidity and mortality data. A commitment to regular and consistent communication between management teams at the local and the regional/national program is critical and may involve the establishment of on-site offices for operational sites in high-risk malarial areas. There should be routine evaluation of areas for improvement, including periodic meetings with senior management to reevaluate the partnership when there are changes in the local epidemiology to warrant modifications [32, 47]. Lastly, formal recognition of partnerships and the shared celebration of program successes are encouraged [33, 35] as well as planning for eventual disappearance of the private company in the area due to several factors such as scarcity of extracted material.

In some cases, the scale of industrial activities demand that local people be relocated. Here, it is best practice to ensure that people are provided newly constructed houses that are built to high standards and that there is improved physical and social infrastructure. In Lao People's Democratic Republic, the construction of the Nam Theun 2 hydroelectric project and planned flooding required the resettlement of 6300 people. The modern wooden homes provided to these people were found to have reduced rates of mosquito house entry compared with traditional houses, and should lead to reduced transmission of malaria and other VBDs [48].

## CHALLENGES OF PRIVATE SECTOR INVOLVEMENT

Although the opportunities are substantial, private sector engagement in malaria control is not without numerous challenges to overcome. In Malaysia, the partnership of the MCP with plantation companies required a significant amount of time from the MCP to ensure that the plantations conducted their agreed activities, while the plantations found it challenging to provide logistical support when vehicles and workers were needed for other purposes [33]. Whilst the health incentive may be compelling, it may also be difficult to provide financial support when there are competing interests, and this is especially true during the development phase of a project compared to the revenue-generating production that follows.

While industrial projects usually consider the potential environmental and social impacts of their activities as part of the permitting or funding process, assessments of health impact often remain voluntary exercises [49]. Health impact assessments (HIAs) can be established as standalone processes or integrated into existing frameworks, but many low- and moderate-income countries lack the regulatory capacity to require these for projects, and studies from Europe indicate that where HIAs are not mandatory, public health can be overlooked [50]. Where HIAs have been used for industrial projects in malaria-endemic areas they have provided a useful contribution for evidence-based decision making [51, 52]. Additional good practice case studies are needed to increase their visibility and advocate for their institutionalization [49, 53]. Further, the World Bank and other funders might adopt more leading roles to ensure that HIAs are conducted in addition to environmental and social assessments. When HIAs are used, they must consider the full range of VBDs that might affect the health of the impacted communities.

Funders can demand that best practices are met through contractual clauses, and may set guidance for the actions of the private sector. A review of the supply chain of the oil and gas industry, where contractors, suppliers, and other indirect employees represent a large portion of the workforce and are at risk of contracting malaria and other communicable diseases, found that contractual clauses on these diseases are becoming more prevalent and are generally considered effective [54]. Funders should increase the prevalence of these clauses to prioritize malaria management, and must have mechanisms for holding companies to account for the health impacts of their practices.

A further challenge for the industry is ensuring that malaria control survives after industrial activities cease. At some point, all resource development projects cease operations, but the long-term planning required for establishing strong enabling partnerships are often lacking. Sustainability planning must begin early in a project’s life, and requires careful consideration for capacity/ability building and the development of realistic time-limited goals.

Gaps in capacity in the technical and programmatic sense, and weak partnerships with limited harmonization at multiple levels, both governmental and with nongovernmental organizations, also impact malaria engagement [46]. Finally, industry-based interventions potentially carry ethical risks [55]. The dilemma comes in the form of resource allocation, with specific groups being protected rather than the general population. Relieving the burden created by malaria requires humanitarian, as well as economically driven, contributions to be successful and sustainable.

None of these potential barriers are unique to resource development projects in low- to moderate-income countries, but overcoming them requires ingenuity, good planning, perseverance, and long-term commitments. In general, the prevailing mandate in the extractive industry is promoting corporate responsibly structures that place an emphasis on all stakeholders, including development-impacted communities near industrial operations [13, 36, 55]. To have a broader impact, industry programs must be part of a larger community of stakeholders with the same goals, rather than operate independently.

## CONCLUSION

Although companies may see the value of investing in national and provincial health and social welfare programs, healthcare is primarily a concern of the government and, therefore, may be viewed as beyond their responsibility and expertise [56]. Nonetheless, private sector industries should have a responsibility and business incentive to provide adequate healthcare and preventive public health services for employees and local communities during development and production phases, and after (in a transitional period to closure) industrial activities cease.

Strengthening inter- and intrasectoral action and collaboration represents the first of 4 pillars that make up the GVCR framework that recognizes that reduction of disease burden through vector control is a shared responsibility of all members of the society. Specifically, the GVCR requires effective coordination of vector control activities between health and nonhealth sectors [45]. The advantages that the private sector offers to health security should be leveraged to ensure that VBD preparedness in resource development settings extends well beyond the institutionalized function, an approach that has been recognized as being more effective in the management of disaster risk [17, 37, 56].

## Notes

***Acknowledgments.*** We thank Florence Fouque and two anonymous reviewers for valuable comments on an earlier version of this manuscript.

***Financial support.*** This work was supported by UNICEF/UNDP/World Bank/WHO Special Programme for Research and Training in Tropical Diseases (TDR). TDR proposed the study through their call for reports on Multi-Sectoral Approaches for The Prevention and Control of Malaria and Emerging Arboviral Diseases: The Impact of Industrial Activities on Vector-Borne Disease Transmission.

***Supplement sponsorship.*** This Supplement is sponsored by TDR: the Special Programme for Research and Training in Tropical Diseases co-sponsored by the United Nations Children’s Fund (UNICEF), the United Nations Development Programme (UNDP), the World Bank and the World Health Organization (WHO).

***Potential conflicts of interest.*** All authors: No reported conflicts of interest. All authors have submitted the ICMJE Form for Disclosure of Potential Conflicts of Interest. Conflicts that the editors consider relevant to the content of the manuscript have been disclosed.

Presented in part: TDR workshop meeting, Geneva, Switzerland, June 2017.
